# Neoadjuvant Imatinib in Recurrent/Metastatic Gastrointestinal Stromal Tumors: A Systematic Review and Meta-analysis of Proportions

**DOI:** 10.1007/s12029-025-01210-2

**Published:** 2025-03-26

**Authors:** Niki Stavrou, Nikolaos Memos, Charalampos Filippatos, Theodoros N. Sergentanis, Flora Zagouri, Maria Gavriatopoulou, Ioannis Ntanasis-Stathopoulos

**Affiliations:** 1 Department of Clinical Therapeutics, School of Medicine, Alexandra Hospital, National and Kapodistrian University of Athens, Athens, Greece; 2 Surgical Department, School of Medicine, Aretaieio Hospital, National and Kapodistrian University of Athens, Athens, Greece; 3 Department of Public Health Policy, University of West Attica, Athens, Greece

**Keywords:** Gastrointestinal stromal tumor, Imatinib, Neoadjuvant, Metastatic, Recurrent, Surgery

## Abstract

**Introduction:**

Metastatic and recurrent gastrointestinal stromal tumors (GISTs) present challenging clinical management. Imatinib is the standard first-line therapy, improving survival and reducing tumor burden in the neoadjuvant use, facilitating surgical intervention. This systematic review and meta-analysis assessed the efficacy of neoadjuvant imatinib in metastatic/recurrent GISTs, highlighting its potential to enhance surgical outcomes and overall patient management.

**Methods:**

A systematic search was conducted in PubMed, Embase and Scopus (end-of-search: February 13, 2025) for records on neoadjuvant imatinib therapy in recurrent/metastatic GISTs. Pooled proportions and 95% confidence intervals were calculated with common-effect and random-effects models. Subgroup and meta-regression analysis were performed, addressing heterogeneity and examining any potential association between the factors that varied and the outcomes reported. The present meta-analysis was performed following the Preferred Reporting Items for Systematic Reviews and Meta-Analysis (PRISMA) guidelines.

**Results:**

The search identified 957 articles, and 14 were analyzed. The meta-analysis of proportions indicated that 2-year and 5-year PFS were 76% (95% CI 58–88%) and 43% (95% CI 17–74%), respectively, while 2-year and 5-year OS were 84% (95% CI 78–89%) and 60% (95% CI 51–68%), respectively. The pooled R0 resection rate was 82% (95% CI 64–92%), associated positively with that of radiological partial response (PR) (*β* = 3.92, *p* < 0.001). Further meta-regression analysis yielded no significant association with preoperative imatinib duration.

**Conclusion:**

The present meta-analysis of trials and studies on metastatic or recurrent GISTs highlights key insights into post-surgery patient outcomes following neoadjuvant treatment with imatinib. Pooled effect estimates revealed promising 2-year and 5-year PFS rates of 76% and 43%, respectively, and 2-year and 5-year OS rates of 84% and 60%, respectively. Furthermore, the high pooled R0 resection rate of 82% emphasizes a substantial surgical efficacy in this population, while it was significantly correlated with successful R0 resections in patients with favorable outcomes.

**Supplementary Information:**

The online version contains supplementary material available at 10.1007/s12029-025-01210-2.

## Introduction

Gastrointestinal stromal tumors (GISTs) represent a small proportion of gastrointestinal cancers, accounting for approximately 1–2% of all malignant tumors in the GI tract [1–3]. Despite their rarity, they are recognized as the most common mesenchymal neoplasms of the gastrointestinal system, distinguishing them from the more frequent epithelial-based adenocarcinomas [4]. GISTs typically arise from the precursors of interstitial cells of Cajal located in the myenteric plexus, which play a crucial role in regulating and sustaining gastrointestinal motility [5]. They are characterized by mutations in receptor tyrosine kinase genes, predominantly the c-KIT gene, which results in uncontrolled cellular proliferation [6, 7]. Clinically, GISTs can cause symptoms such as abdominal pain, gastrointestinal bleeding, and obstruction, which may lead to delayed diagnosis [8, 9]. Surgical resection is the primary treatment for localized GISTs, aiming for an R0 resection to ensure all tumor cells are removed [9].

For metastatic and recurrent GISTs, targeted therapies are crucial. Imatinib is the standard first-line treatment, especially for tumors with KIT exon 11 mutations, typically administered at a dose of 400 mg/day is recommended, with a potential escalation to 800 mg/day for more aggressive mutations [10, 11]. It is a highly selective inhibitor of various protein kinases, including BCR-ABL, platelet-derived growth factor receptors, and c-KIT. Since the introduction, survival rates for patients with advanced or metastatic GISTs [10] have significantly improved, with median overall survival (OS) now exceeding 5 years in many studies, compared to only 1.5 years prior to imatinib’s use [12–14]. Patients harboring KIT exon 11 mutations tend to respond better, showing higher response rates and longer progression-free survival (PFS) than those with other mutations [15].

Surgical intervention for metastatic as well as recurrent GISTs, particularly in patients already on imatinib, can be considered in managing disease progression. When patients exhibit localized metastatic/recurrent disease that is amenable to resection, surgery can significantly enhance survival rates, even after systematic therapy has begun. Studies indicate that imatinib may reduce tumor burden and facilitate resection, emphasizing the importance of a multidisciplinary approach to evaluate surgical options in this context [16–19].

In this context, we conducted a comprehensive systematic review and meta-analysis of studies and clinical trials on recurrent/metastatic GISTs to assess the efficacy of neoadjuvant imatinib therapy, highlighting its potential to enhance surgical outcomes and overall patient management.

## Materials and Methods

The present meta-analysis was performed following the Preferred Reporting Items for Systematic Reviews and Meta-Analysis (PRISMA) guidelines [20]. The study protocol was discussed and agreed upon in advance by all authors, but it was not registered in an online review registry.

A systematic search was conducted in the PubMed, Embase, and Scopus databases from inception until February 13, 2025, to identify peer-reviewed and full-text articles on the use of neoadjuvant imatinib in recurrent/metastatic GIST. The search algorithm used “(GIST OR (GASTROINTESTINAL STROMAL TUMORS) OR (GASTROINTESTINAL STROMAL TUMOURS) OR (GASTROINTESTINAL STROMAL NEOPLASM)) AND (RECURRENT OR METASTATIC) AND (PREOPERATIVE OR NEOADJUVANT) AND (IMATINIB OR GLEEVEC OR GLIVEC)” was ensured to adhere to the databases’ unique characteristics, implementing variations of key terms. Additionally, a comprehensive and systematic snowball approach was used to capture all relevant records by reviewing the reference lists of included studies, ensuring comprehensive coverage and minimizing the risk of omitting previously cited literature [21].

Eligible studies included clinical trials (randomized or not) and prospective or retrospective studies focusing on adults diagnosed with recurrent/metastatic GIST that were treated with imatinib prior to surgery. Exclusion criteria encompassed case reports, case series, reviews, in vitro and animal studies, and records not available in English.

### Data Extraction and Effect Estimates

The data extraction encompassed general information (first author’s name, publication year, database ID), study characteristics (design, cohort size, follow-up, geographic region, number of males, age), population characteristics (location of primary malignancy, site of metastases, number and size of lesions, KIT mutations), intervention characteristics (preoperative imatinib therapy duration, preoperative imatinib dosage) and outcomes overall survival (OS), progression-free survival (PFS), resection rates (R0 for microscopically complete resection, R1 for microscopically incomplete resection, and R2 for macroscopically incomplete resection), and RECIST criteria assessing the radiological response (CR for complete response, PR for partial response, SD for stable disease, and PD for progressive disease). Extracted effect estimates included crude numbers and percentages for the outcomes alongside their 95% confidence intervals (CI). In case the aforementioned data were not available in the main text, the supplementary material was thoroughly screened in order to extract or reproduce the corresponding omitted results [22]. There was no shortage of required data for the purposes of the meta-analysis. Data were independently extracted, analyzed, and recorded. The finalized data form was reached after team consensus.

### Statistical Analysis

Extracted data for continuous numerical variables such as age and follow-up, reported in the original studies as either means or medians, were standardized to means using the method proposed by Hozo et al. [23]. This conversion ensured consistency across data points, allowing for uniformity in their use for statistical analysis.

An overall analysis of proportions of OS and PFS at 2-year and 5-year timepoints and R0/R1/R2 resections was chosen as the base-case analysis to evaluate the effect of preoperative therapy with imatinib in patients with recurrent/metastatic GIST. When proportions of OS and PFS at 2-year and 5-year timepoints were unavailable, these were either extracted from published Kaplan–Meier survival curves or calculated directly from patient outcome data provided in the eligible records.

Statistical analysis included pooling of studies as well as post meta-regressions. Common and random effects models were appropriately used to calculate the pooled effect estimates (proportions). Study heterogeneity was assessed by *Q*-test and *I*^2^ estimations. When heterogeneity was not low (*I*^2^ and *Q*-test conclusions), random-effect model results were deemed appropriate. Subgroup analysis was performed based on design and geographic region in case of more than 5 entries per group. Post hoc meta-regression analysis was performed to assess whether other moderators within the study sample modified the reported effect estimates. Variables included were prespecified key study aspects from the extracted data that introduced heterogeneity and had 10 or more entries.

Across this analysis, *I*^2^ < 40% or *p* (*Q*-test) < 0.10 was considered low heterogeneity, and statistical significance was achieved by *p*-values < 0.05.

All statistical analyses were performed using R/R-Studio version 2024.04.2 + 764) (Posit Software, PBC).

### Assessment of Study Quality and Risk of Bias

All records included clinical trials, prospective or retrospective cohorts, or case–control studies. The risk of bias was assessed with the implementation of RoB 2 algorithm by Cochrane and the Newcastle–Ottawa scale to our analysis tools [20, 24].

A publication bias assessment was decided not to be conducted in this study, as in such cases of meta-analysis of proportions, it has been associated with misleading results, and its use is not recommended [25, 26].

## Results

**Fig. 1:**
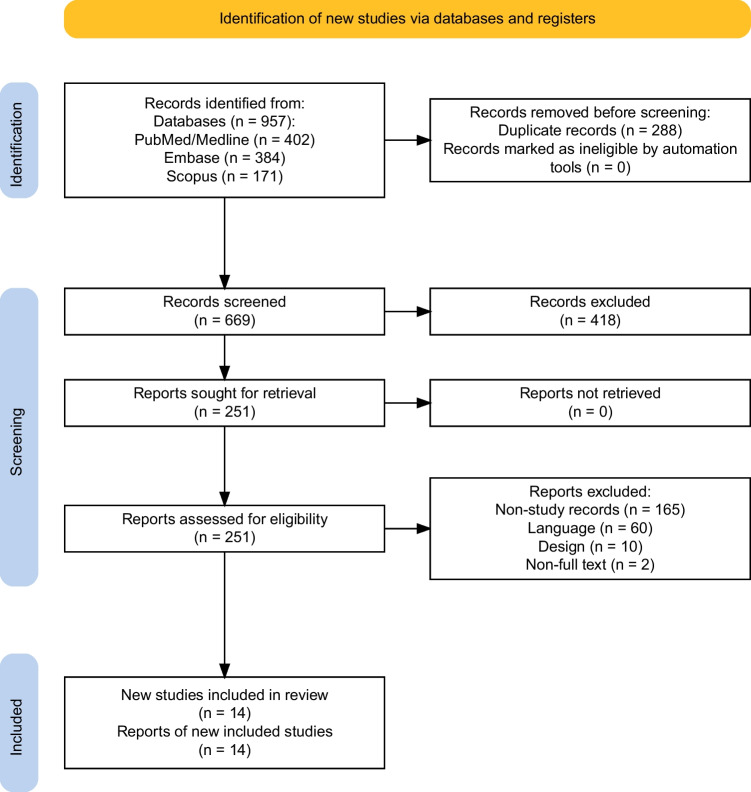
PRISMA 2020 flowchart of study selection

Nine hundred and fifty-seven records were identified by utilizing the pre-specified search algorithm over the aforementioned databases. Two hundred and eighty-eight records were removed before screening, as they were duplicates. Each article underwent an evaluation for relevance to the designated topic, leading to exclusion of 418 publications that were deemed irrelevant, per initial review. The remaining 251 reports were screened in accordance with the eligibility criteria outlined in the “Materials and Methods” section. This comprehensive review resulted in the exclusion of 165 non-study records, 60 not available in English, 10 due to study design, and 2 as they were not full-text and peer-reviewed articles. The following PRISMA flowchart depicts the successive steps in the selection of studies, which ultimately resulted in 14 articles that were included in the final analysis [27–41] (Fig. 1).

These 14 records reported results from 1 prospective study, 10 retrospective studies, and 3 clinical trials involving 328 patients with recurrent/metastatic GISTs that were treated with imatinib prior to surgery. Descriptive characteristics of the included studies are portrayed in Table 1, and more information is provided in Supplementary Table 4.

**Table 1:** Descriptive characteristics of the included studies

Study	Design	Region	Patients (*n*^a^)	Males (%)	Age (years)	Median preoperative IM^b^ duration (months)	Preoperative IM dosage (mg/d)^d^
Qi et al. (2020)	Retrospective	Asia	7	85.7	47	8	400–600
Wang et al. (2020)	Retrospective	Asia	12	91.7	56.2	11.4	400
Chen et al. (2019)	Retrospective	Asia	15	60.0	53	10	400–800
Roland et al. (2018)	Retrospective	USA	87	54.0	55	22.2	-
Ramaswamy et al. (2014)	Retrospective	Asia	9	-	-	-	-
Cananzi et al. (2014)	Retrospective	EU	11	36.4	51	38	-
Shen et al. (2014)	Prospective	Asia	5	80.0	42	8	400–600
Bednarski et al. (2014)	Retrospective	USA	53	67.9	59	17.9	-
Du et al. (2014)	Clinical trial	Asia	19	57.9	49	6.3	400
Wang et al. (2013)	Retrospective	Asia	22	59.1	49.3	14	400–800
Wang et al. (2012)	Clinical trial	Global	22	59.1	53	2.1	600
Xia et al. (2010)	Clinical trial	Asia	19	52.6	53	6	600
Andtbacka et al. (2007)	Retrospective	USA	35	-	55.7	15.2	400–800
Bauer et al. (2005)	Retrospective	EU	12	50.0	60.0	12.2	400–600

^a^*n* number of patients, ^b^*IM* imatinib, ^d^*mg/d* milligrams per day

### 2- and 5-Year PFS Rates

**Fig. 2:**
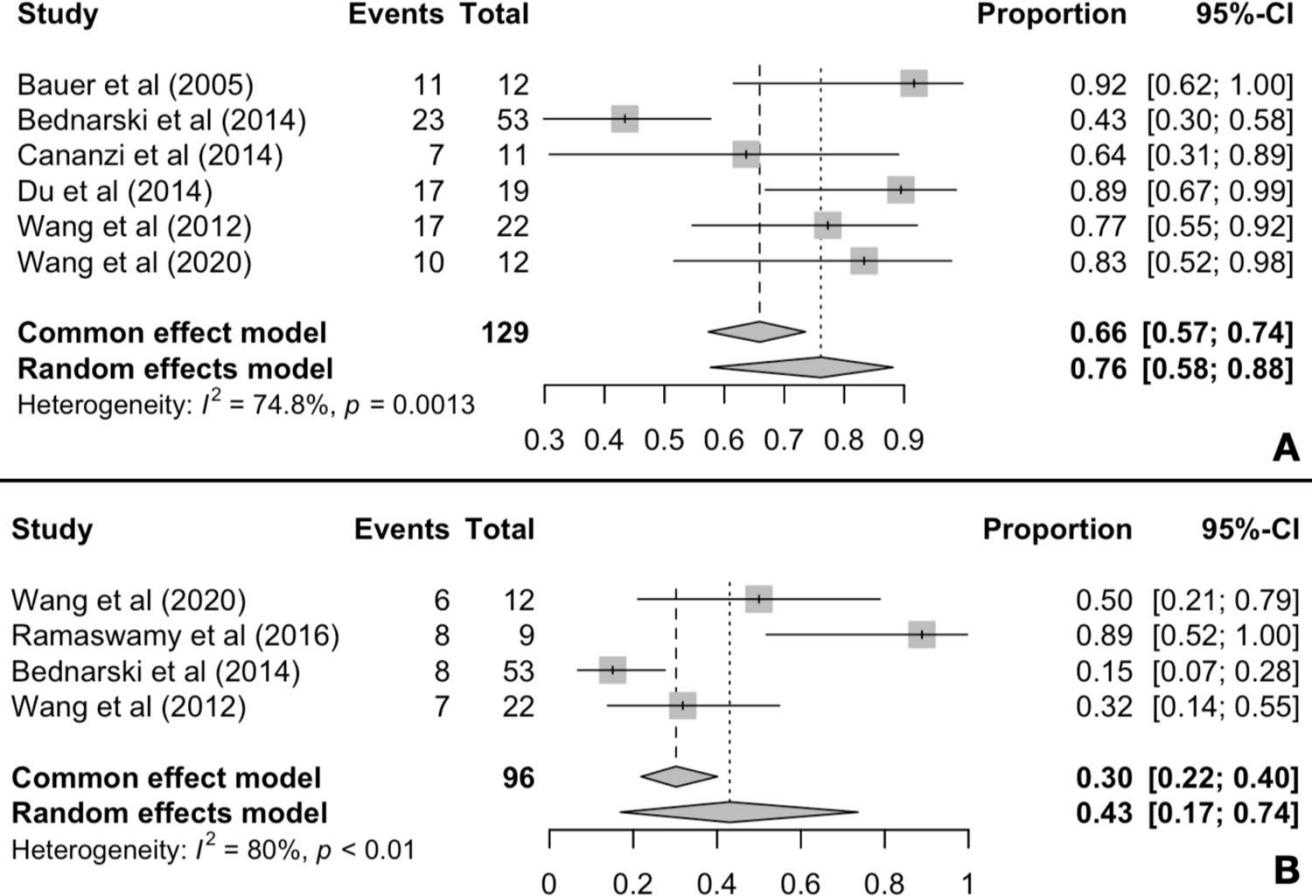
Pooled 2-year PFS (**A**) and 5-year PFS (**B**) rates

A total of 6 out of the 14 studies reported PFS data at the 2-year timepoint, including 129 patients [28, 32, 34, 35, 37, 41]. Meta-analysis results indicate that 76% (95% CI 58–88%) of the patients treated with imatinib prior to surgery survive 2 years after surgery without exhibiting disease progression. The pooling of the reported PFS outcomes demonstrated high heterogeneity (*I*^2^ = 74.8%, *p* < 0.01) (Fig. 2A).

Furthermore, 4 studies out of the 14 included reported PFS rates at the 5-year timepoint, including 96 patients [28, 31, 34, 37]. The meta-analysis of proportions resulted at a 43% (95% CI 17–74%) 5-year PFS rate while heterogeneity was high (*I*^2^ = 80%, *p* < 0.01) (Fig. 2B).

### 2- and 5-Year OS Rates

**Fig. 3:**
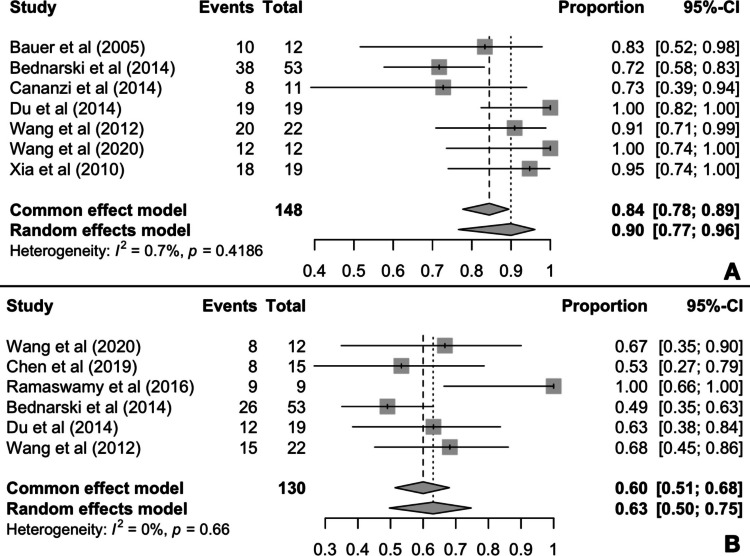
Pooled 2-year OS (**A**) and 5-year OS (**B**) rates

Seven out of the 14 studies included reported 2-year OS rates, encompassing a total of 148 patients [28, 32, 34, 35, 37, 38, 41]. Meta-analysis results indicate that 84% (95% CI 78–89%) of the patients treated with imatinib prior to surgery survived 2 years of post-operation. Given the almost negligible heterogeneity observed (*I*^2^ = 0.7%, *p* = 0.42), the interpretation of the common-effect model was deemed appropriate (Fig. 3A).

Furthermore, 6 out of the 14 included studies reported OS data at the 5-year timepoint [28, 29, 31, 34, 35, 37]. Pooled proportion analysis revealed a 60% (95% CI 51–68%) 5-year OS rate.

Heterogeneity was once again particularly low (*I*^2^ = 0%, *p* = 0.66), allowing for the interpretation of the common-effect model estimates (Fig. 3B).

### Microscopically Complete Resection (R0) After Neoadjuvant Imatinib Therapy

**Fig. 4:**
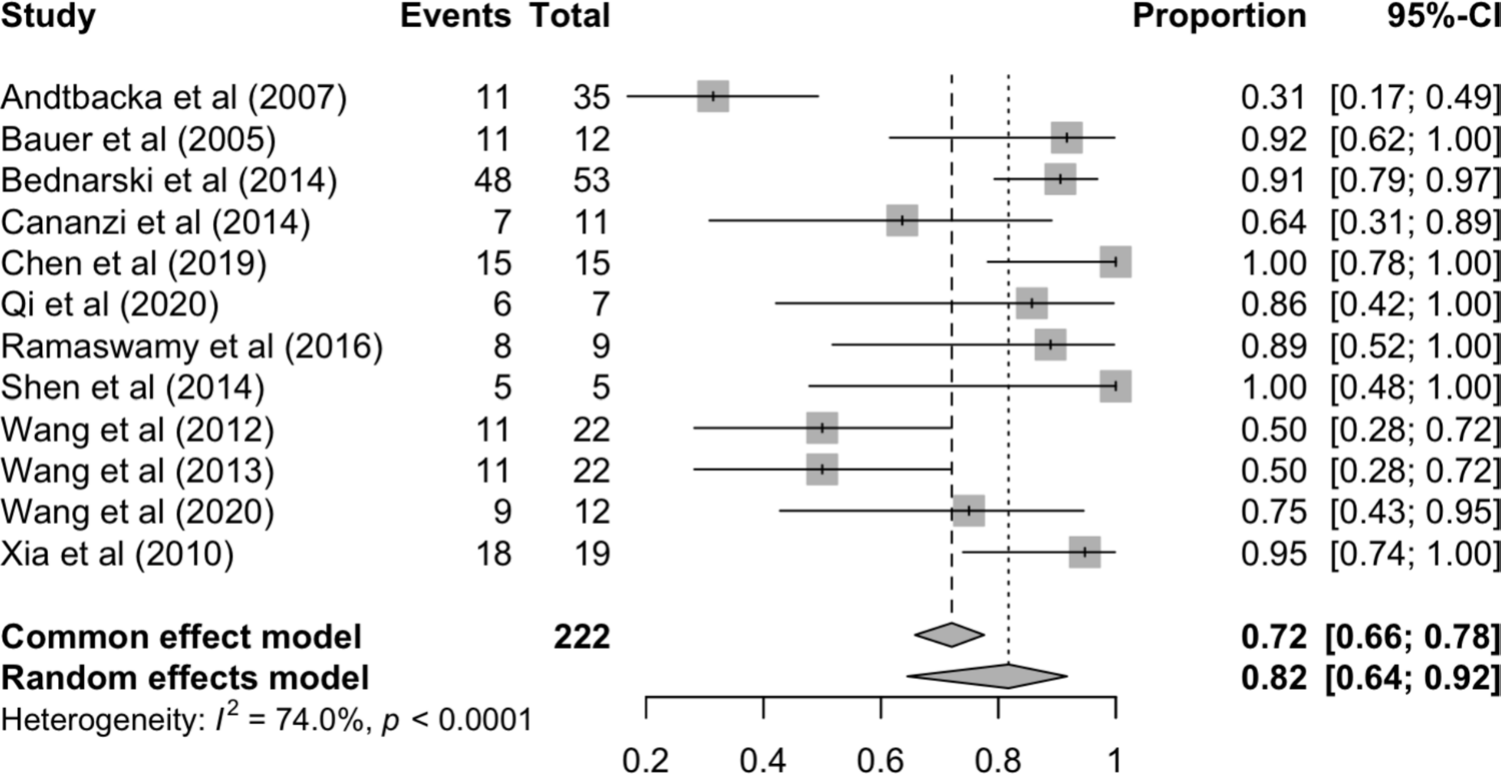
Pooled R0 resection rate

The meta-analysis of proportions of R0 in 222 patients across 12 studies [27–29, 31–34, 36–39, 41] yielded a pooled overall success rate of 82% (95% CI 64–92%) for microscopically complete resections after neoadjuvant imatinib therapy (Fig. 4). A high level of heterogeneity is demonstrated (*I*^2^ = 74%, *p* < 0.01) across the reported R0 resection outcomes, while subgroup analysis by study design and geographic region revealed no statistically significant and marginally significant differences between groups, respectively (Supplementary Figs. 1 and 2).

**Fig. 5:**
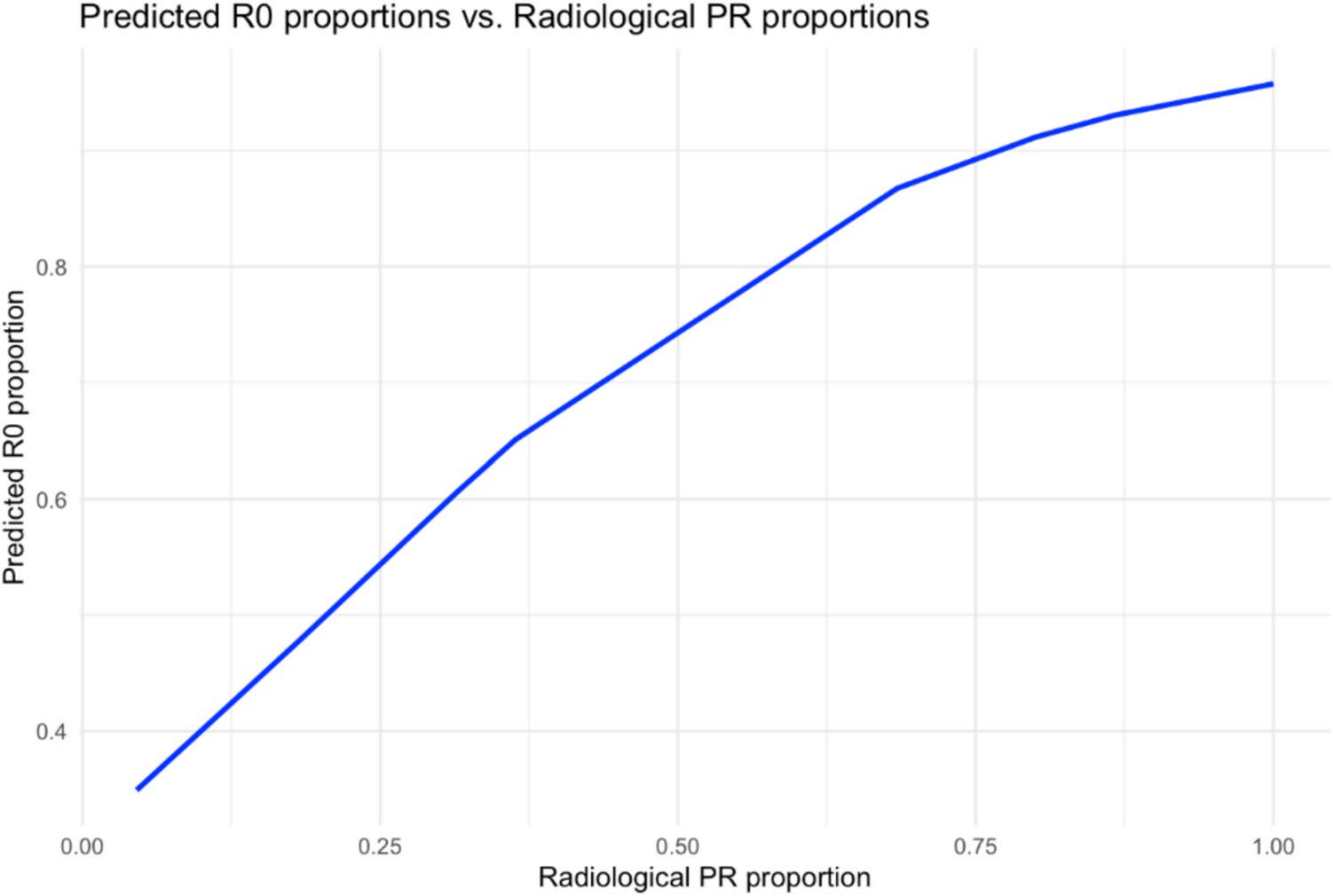
Meta-regression fit curve of association between R0 and radiological PR proportions

Post hoc meta-regression analysis revealed a strongly statistically significant and positive association between the proportion of patients achieving radiological PR and the proportion of R0 resections (*α* =  − 0.80, *β* = 3.92, *p* < 0.001) (Supplementary Table 1). More specifically, a linear association on the logit scale was translated to a near-linear association on the proportion scale, flattening out close to max values (Fig. 5). The negative intercept *α* =  − 0.80 denotes a negative association when the moderate is absent, in this case, when a radiological PR is not achieved. The coefficient (*β* = 3.92) means that, for each percentage point increase (1% increase) in radiological PR, the log-odds of R0 resection increase by approximately 0.0392 times. Moreover, the proportions of radiological PRs explained the rate of 25% of the initial heterogeneity (initial *I*^2^ = 74%, new *I*^2^ = 49%).

### Microscopically and Macroscopically Incomplete Resections (R1 and R2) After Neoadjuvant Imatinib Therapy

Proportions of R1 and R2 resections were available from 6 [28, 30, 35–38] and 9 studies [27, 28, 31, 34–39], involving 181 and 198 patients, respectively. Meta-analysis of proportions resulted in 35% (95% CI 2–93%) and 16% (95% CI 7–32%) for R1 and R2 resection rates, respectively (Supplementary Figs. 3 and 4).

### Risk of Bias

Both retrospective and prospective studies included were assessed by the Newcastle–Ottawa assessment scale [22] and characterized according to score as high- or low-quality studies. Three studies were of low quality (score = 6), and nine studies were of high quality (score = 7) (Supplementary Table 2). The RoB 2 algorithm by Cochrane was utilized for the two randomized clinical trials included and resulted in some concerns, mainly in the measurement of the reported outcome (Supplementary Table 3).

## Discussion

In our investigation, we identified 2-year and 5-year PFS rates at 76% (95% CI 58–88%) and 43% (95% CI 17–74%), respectively, as well as 2-year OS at 84% (95% CI 78–89%), which subsequently diminished to 5-year OS at 60% (95% CI 51–67%). Also, the R0 resection rate was 82% (95% CI 64–92%), and it was strongly related to tumor response, according to RECIST criteria [42]. It is noteworthy that R0 resection rate was not associated with preoperative IM duration.

This meta-analysis exclusively considers patients with metastatic and/or recurrent GISTs, distinguishing it from the majority of previously published studies that have combined both locally advanced and metastatic/recurrent cases. A recent meta-analysis conducted by Lam et al. [43] evaluated a heterogeneous cohort of both locally advanced and metastatic GISTs, reporting a commendable R0 resection rate of 88.9% (95% CI 84–93.2%), alongside OS rates of 100% (99.2–100%), 94% (95% CI 89.7–98%), and 87.6% (95% CI 78.7–94.6%) at the 1-year, 3-year, and 5-year intervals, respectively. The extant literature elucidates that individuals with locally advanced GISTs typically manifest more favorable survival trajectories compared to their counterparts with metastatic or recurrent disease [25]. In particular, Gheorghe et al. [2] documented a remarkable 5-year life expectancy of 80% for patients with locally advanced GISTs, contrasted with a mere 55% survival rate among those afflicted by metastatic disease.

Furthermore, several reviews have been published regarding survival outcomes of metastatic/recurrent GISTs under first-line treatment with imatinib. For example, Ford et al. [42] supported an operation in a metastatic setting in those patients responding to imatinib or having a limited focal progression, resulting in limited elevation of PFS and OS. Patel [19] summarized survival outcomes from major trials which were designed to study survival outcomes in metastatic and unresectable GISTs under first-line imatinib. An open-label phase III trial of Blanke et al. [43] reported PFS and OS rates according to daily imatinib dosage between 400 and 800 mg. They published that 2-year PFS was 41% and 46% of those who were treated by 400 mg/day and 800 mg/day of imatinib, respectively. Accordingly, 2-year OS was 76% and 72% for patients at 400 mg/day and those at 800 mg/day. Blanke et al., in another trial [44] in a phase II randomized study, reported a 5-year OS rate close to 50%. Another study by Serano et al. reported that 2-year PFS in metastatic patients treated with 400 mg of imatinib per day was 52% and in the escalated dose of 800 mg/day was 44% (HR 0.78). On the other hand, Blay et al. [45] presented more favorable survival results when they studied non-metastatic advanced GISTs and represented the results in a few relevant studies (3-year PFS = 92%, 5-year PFS = 92%) [46–52].

Wang et al. [36] reported findings from the RTOG 0132/ACRIN 6665, a prospective phase II trial that assessed neoadjuvant imatinib therapy for metastatic and recurrent GISTs involving 22 patients (Group B) with a median follow-up of 5.5 years. They observed that the 2-year and 5-year overall survival rates were 90.9% (95% CI 78.9–100%) and 68.2% (95% CI 46.9–89.5%), respectively, while the estimates for 2-year and 5-year PFS were 77.3% (95% CI 59.8–94.8%) and 29.8% (95% CI 8.8–50.7%), respectively. In the same trial, Eisenberg et al. [39] reported an R0 resection at 58% in Group B. Additionally, Wang et al. [36] conducted a further analysis on R status and found no apparent correlation between R status and tumor progression. Bednarski et al. [33] conducted a retrospective analysis of 53 patients and reported findings that align closely with our analysis concerning overall survival (OS) and R0 resection rates. They found that the 2-year OS was 74% (95% CI 60–85%) and the 5-year OS was 49% (95% CI 35–63%). Additionally, they reported an R0 resection rate of 91% (95% CI 79–96%).

In the process of evaluating our analysis results, we noted that the 2-year PFS rate observed in our study of 76% (95% CI 58–88%) exhibited substantial heterogeneity, as indicated by an *I*^2^ value of 75% (*p* < 0.01). This suggests considerable variability in the results across the studies incorporated in the analysis [28, 32]. Similarly, the examination of R0 resection rates also revealed a significant degree of heterogeneity, characterized by an *I*^2^ value of 74% (*p* < 0.01) [27, 28, 31–33]. These findings imply that clinical outcomes may differ markedly based on the specific populations and methodologies employed in the individual studies. The elevated levels of heterogeneity observed in both the PFS and R0 resection rates underscore the imperative for further investigations aimed at elucidating the factors that may influence survival outcomes and R0 rates in patients diagnosed with GISTs. This significant heterogeneity among some of the key outcomes introduces a limitation to our study.

Moreover, it is crucial to acknowledge the rarity of the condition investigated in this study, leading to not only the scarcity of peer-reviewed, full-text publications addressing this matter but also to a low number of patients enrolled in each study or trial, thus resulting in a low overall number of patients involved in this meta-analysis. It is also worth mentioning that the nature of this analysis of proportions inherently introduces a limitation, as there are no control groups in order to compare different strategies, despite drawing safe conclusions. Additionally, the limited number of studies reporting survival outcomes at fixed timepoints renders further subgroup or meta-regression analysis not possible, as they can lead to unreliable or unstable estimates, particularly due to small sample sizes and the aforementioned high variability. This is a limitation of our study, as potential factors that influence heterogeneity were not possible to be evaluated with statistical rigor.

Despite these limitations, this systematic review and meta-analysis boasts several strengths. First, the study adheres to the PRISMA guidelines, ensuring transparency, reproducibility, and methodological rigor. The comprehensive and exhaustive screening of three key databases, combined with a carefully constructed search algorithm and a methodologically sound snowball approach, ensures that no eligible studies are omitted. Second, robust statistical methods are applied throughout the study, leading to reliable effect estimates and thus, conclusions. Finally, this meta-analysis provides valuable clinical and research insights on a niche subgroup of patients, those with recurrent or metastatic GISTs.

## Conclusion

In conclusion, our extensive review of 957 articles across three databases, with the analysis of 14 pertinent studies involving 328 patients with metastatic or recurrent GISTs, highlights key insights into post-surgerical patient outcomes following neoadjuvant treatment with imatinib. The meta-analysis revealed promising 2-year and 5-year PFS rates of 76% and 43%, respectively, and 2-year and 5-year OS rates of 84% and 60%, respectively. Furthermore, the high pooled R0 resection rate of 82% emphasizes a substantial surgical efficacy in this population, while it was significantly correlated with successful R0 resections in patients with favorable outcomes.

## Supplementary Information

Below is the link to the electronic supplementary material.

Supplementary file1 (DOCX 924 KB)

## Data Availability

Supplementary data are available in the supplementary material. Further data is available upon reasonable request from the corresponding author.
