# Supplementary material for: Neoadjuvant Imatinib in Recurrent/Metastatic Gastrointestinal Stromal Tumors: A Systematic Review and Meta-analysis of Proportions
Source: J Gastrointest Cancer. 2025 Mar 26;56(1):88. doi: 10.1007/s12029-025-01210-2 (PMC11947046; doi:10.1007/s12029-025-01210-2)
Supplement: Supplementary file 1 — Supplementary file1 (DOCX 924 KB) [file 12029_2025_1210_MOESM1_ESM.docx]

**Supplementary Figure 1.** Pooled R0 resection rates, subgroup analysis by geographic region


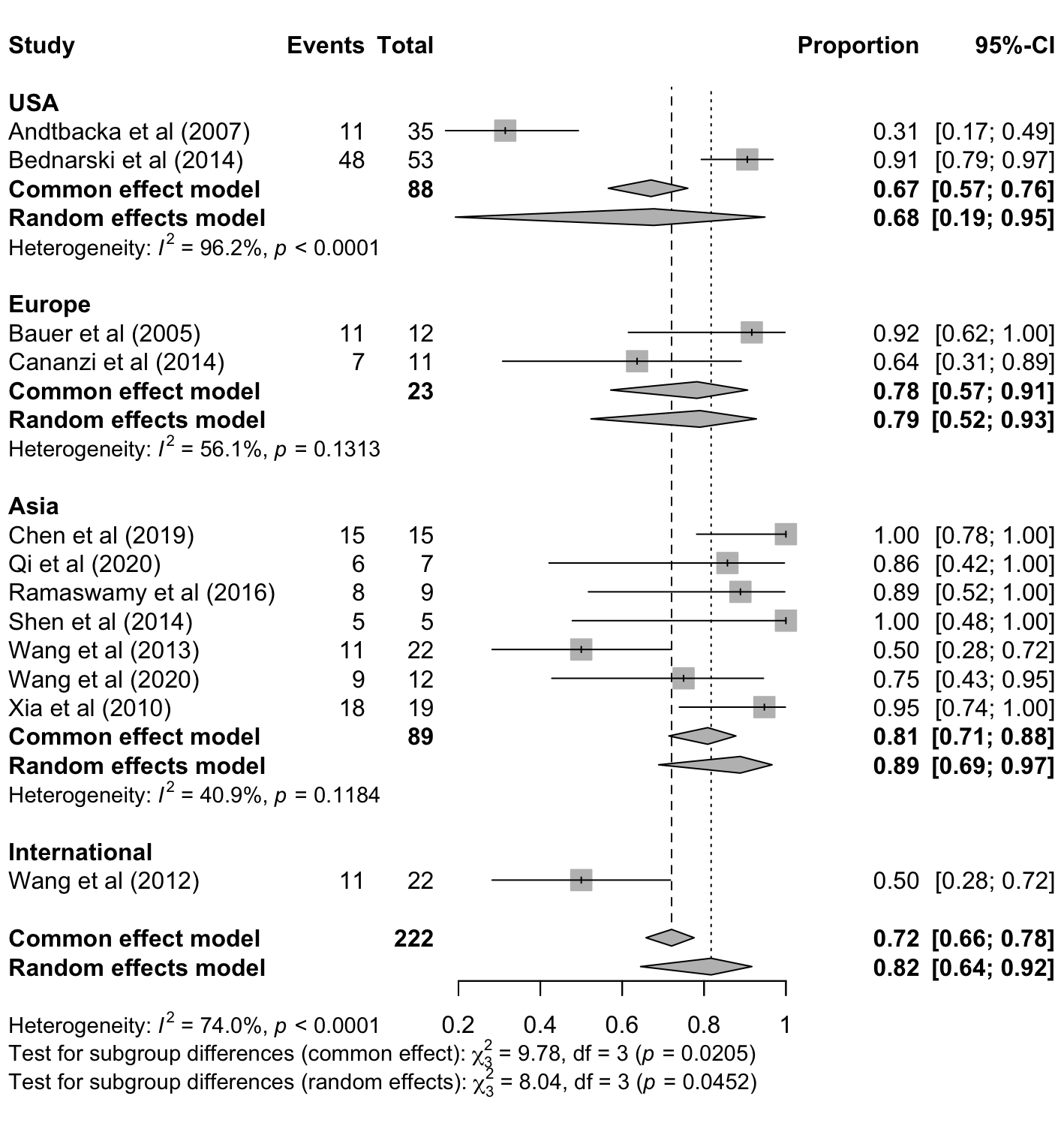


**Supplementary Figure 2.** Pooled R0 resection rates, subgroup analysis by study design


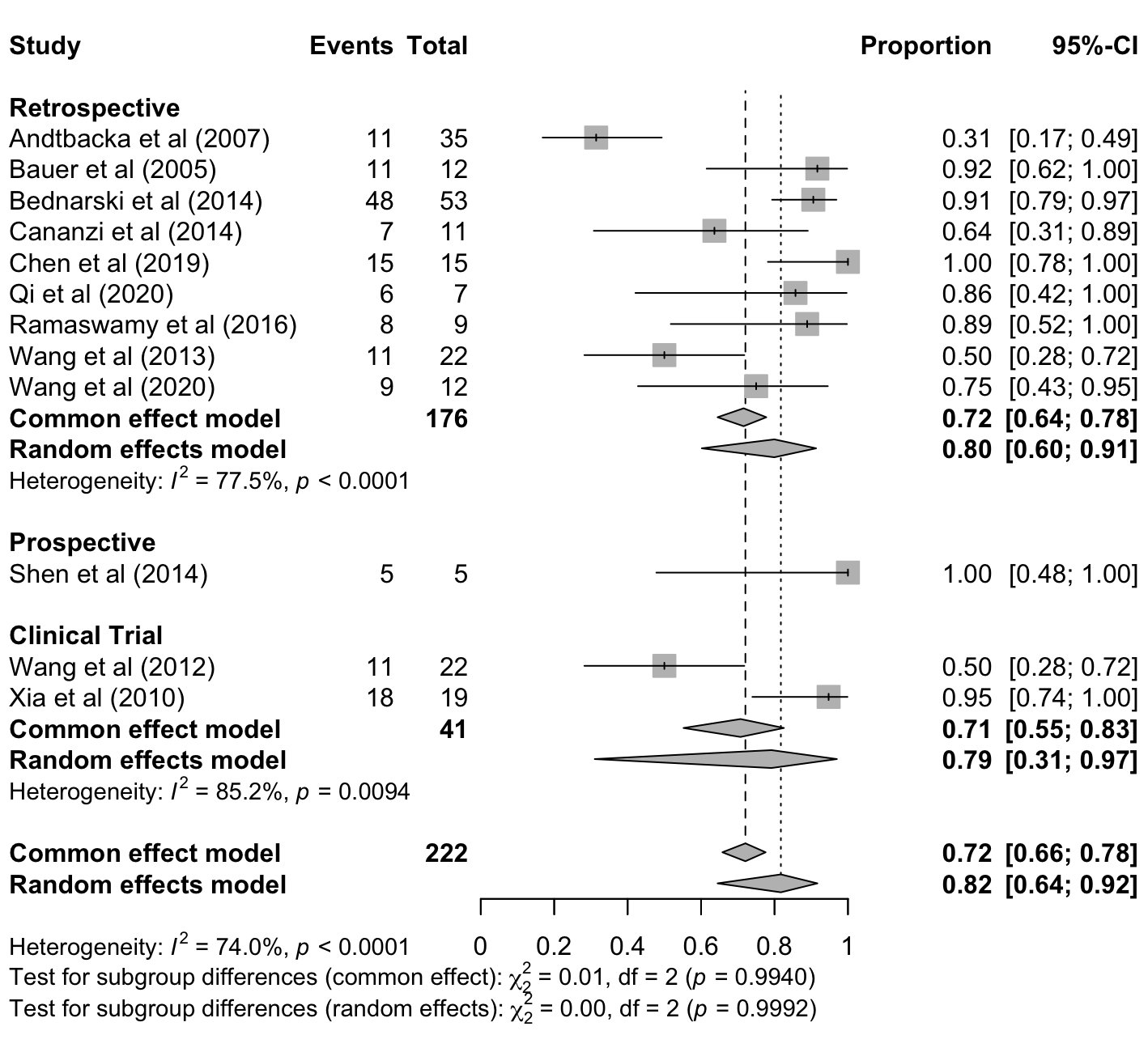


**Supplementary Table 1** Meta-regression results for R0 resection proportions

| **Variable** | **Estimate (Logit scale)** | **p-value** |
| --- | --- | --- |
| Age (median) | -0.02 | 0.672 |
| # of patients | -0.03 | 0.420 |
| % of males | 0.02 | 0.194 |
| Preoperative IM duration (months, median) | -0.03 | 0.514 |
| Preoperative IM dosage (mg)  600  400-600  400-800 | 0.11  1.59  -0.53 | 0.948  0.372  0.742 |
| % of Radiological PR | 3.92 | <0.001*** |

**Supplementary Figure 3.** Pooled R1 resection rates


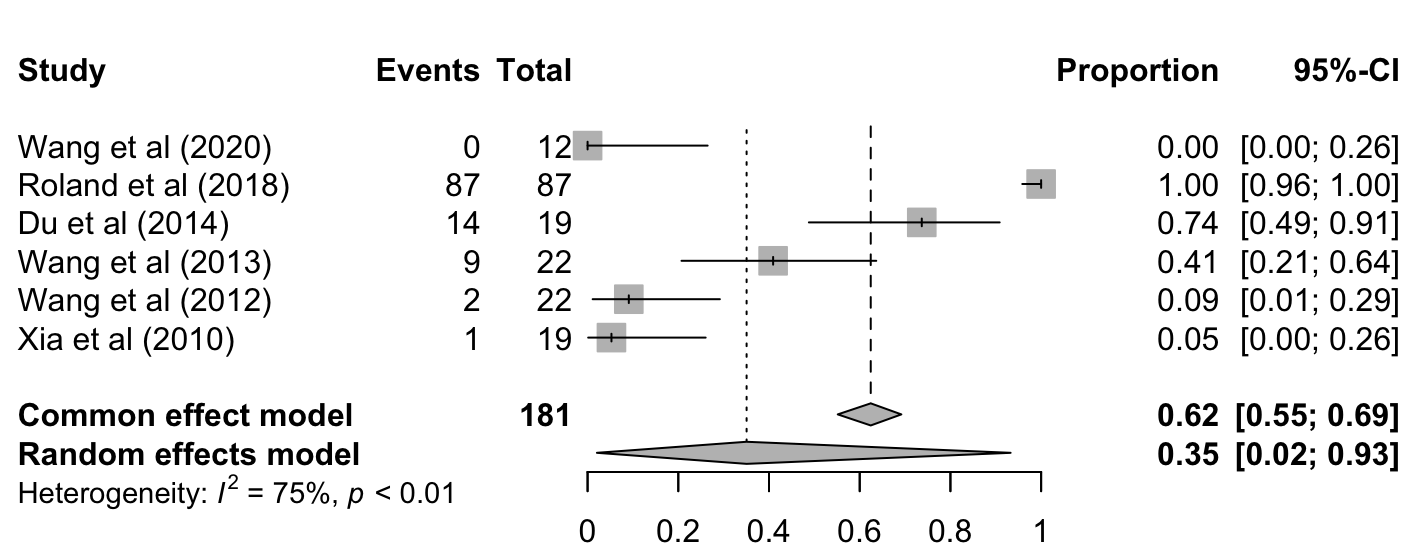


**Supplementary Figure 4.** Pooled R2 resection rates


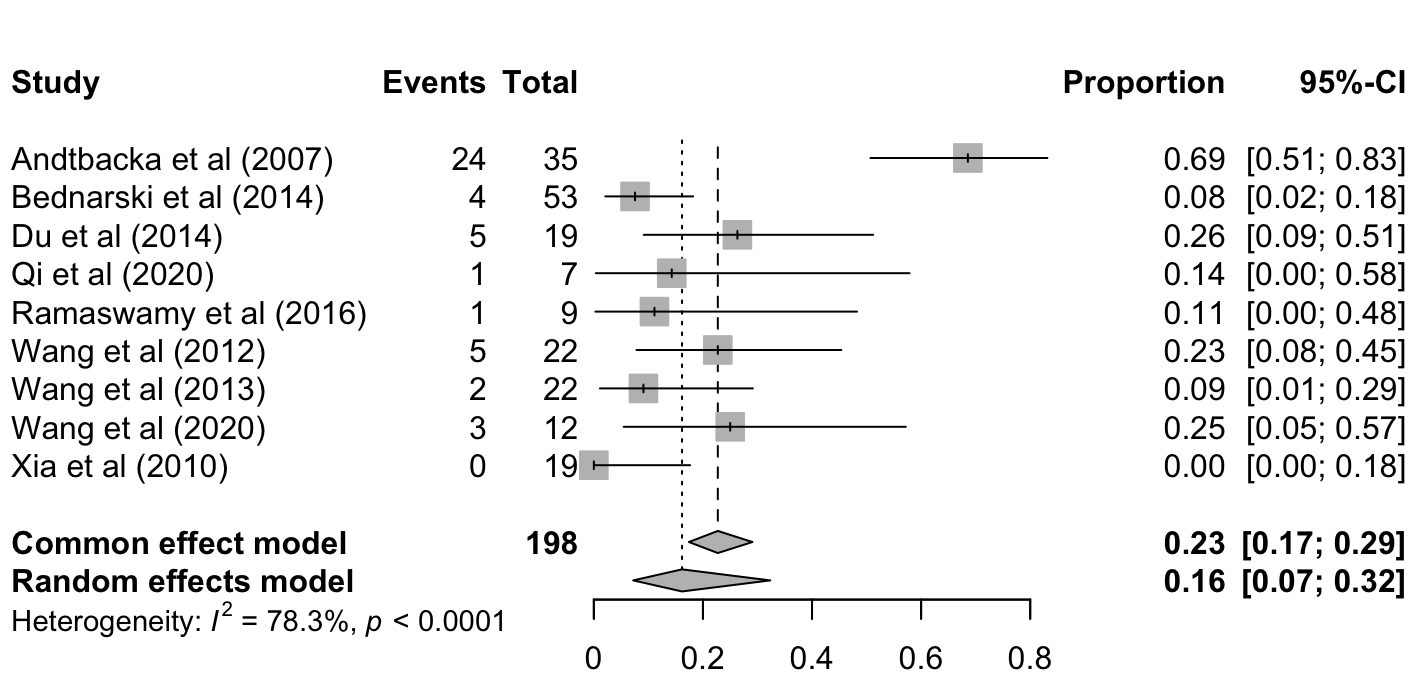


**Supplementary Table 2.** Risk of bias assessment for non-randomized studies

| STUDY | SELECTION | COMPARABILITY | OUTCOME/  EXPOSURE | NUMBER OF  STARS |
| --- | --- | --- | --- | --- |
| Qi et al (2020) | ****** | ****** | ****** | 6 |
| Wang et al (2020) | ****** | ****** | ****** | 6 |
| Chen et al (2019) | ****** | ****** | ******* | 7 |
| Roland et al (2018) | ****** | ****** | ******* | 7 |
| Ramaswamy et al (2014) | ****** | ****** | ******* | 7 |
| Cananzi et al (2014) | ****** | ****** | ******* | 7 |
| Shen et al (2014) | ****** | ****** | ****** | 6 |
| Bednarski et al (2014) | ****** | ****** | ******* | 7 |
| Wang et al (2013) | ****** | ****** | ******* | 7 |
| Wang et al (2012) | ****** | ****** | ******* | 7 |
| Andtbacka et al (2007) | ****** | ****** | ******* | 7 |
| Bauer et al (2005) | ***** | ****** | ******* | 7 |

**Supplementary Table 3.** Risk of bias assessment for randomized controlled trials

| **Author (year)** | **Randomization process** | **Deviations from intended interventions** | **Missing outcome data** | **Measurement of the outcome** | **Selection of the reported result** | **Overall bias** |
| --- | --- | --- | --- | --- | --- | --- |
| Du et al (2014) | Low | Low | Low | Some concerns | Low | Some concerns |
| Xia et al (2010) | Low | Low | Low | Some concerns | Low | Some concerns |

| **Study** | **Design** | **Region** | **N** | **Males (%)** | **Age (years)** | **Median FU**  **(mo)** | **Primary site**  **Stomach**  **S.I.**  **L.I.**  **Other** | **Site of**  **metastasis**  **Liver**  **Peritoneum**  **Other** | **Preoperative**  **IM duration (mo)** | **Preoperative**  **IM**  **dosage**  **(mg/d)** | **KIT mutation** | **Lesions**  **(=1)**  **(>=2)** |
| --- | --- | --- | --- | --- | --- | --- | --- | --- | --- | --- | --- | --- |
| Qi et al (2020) | R | Asia | 7 | 85.7 | 47 | 55.12 | 3  2  0  2 | 1  -  2 | 8 | 400-600 | Exon 11: 5  Exon 9: 2 | - |
| Wang et al (2020) | R | Asia | 12 | 91.7 | 56.2 | 39.9 | 3  3  2  4 | -  -  - | 11.4 | 400 | - | - |
| Chen et al (2019) | R | Asia | 15 | 60.0 | 53 | 26 | 5  -  -  10 | 15  0  0 | 10 | 400-800 | - | -  12 |
| Roland et al (2018) | R | USA | 87 | 54.0 | 55 | 51 | 33  38  10  6 | 27  24  36 | 22.2 | - | Exon 11: 22  Exon 9: 4  Wild-type: 8  Exon 2: 6  Other: 6  Unknown: 41 | 24  63 |
| Ramaswamy et al (2014) | R | Asia | 9 | - | - | 24 | 6  1  2  0 | -  -  - | - | - | - | - |
| Cananzi et al (2014) | R | EU | 11 | 36.4 | 51 | 65 | 5  -  -  6 | 11  -  - | 38 | - | - | 4  7 |
| Shen et al (2014) | P | Asia | 5 | 80.0 | 42 | 9.5 | 0  3  2  0 | 2  -  3 | 8 | 400-600 | Exon 11: 2  Exon 9: 2  Unknown: 1 | - |
| Bednarski et al (2014) | R | USA | 53 | 67.9 | 59 | 28.8 | 16  29  4  3 | -  -  - | 17.9 | - | Exon 11: 28  Exon 9/13/17: 7  Wild-type: 6 | - |
| Du et al (2014) | CT | Asia | 19 | 57.9 | 49 | 23 | 5  10  2  2 | -  -  - | 6.3 | 400 | - | 10  9 |
| Wang et al (2013) | R | Asia | 22 | 59.1 | 49.3 | 53 | 7  7  4  4 | 3  2  17 | 14 | 400-800 | Exon 11: 12  Exon 9: 4  Exon 13: 1  Wild-type: 1  PDGFRA ex18 D842Vmut: 4 | - |
| Wang et al (2012) | CT | Global | 22 | 59.1 | 53 | 66 | -  2  -  - | 7  10  3 | 2.1 | 600 | - | - |
| Xia et al (2010) | CT | Asia | 19 | 52.6 | 53 | 36 | 11  5  1  2 | 19  -  - | 6 | 600 | Exon 11: 7  Exon 9: 3  Unknown: 8 | 7  12 |
| Andtbacka et al (2007) | R | USA | 35 | - | 55.7 | 36.2 | -  -  -  - | 7  -  - | 15.2 | 400-800 | - | 9  23 |
| Bauer et al (2005) | R | EU | 12 | 50 | 60 | 29.8 | 5  5  -  2 | 9  5  1 | 12.2 | 400-600 | - | 9  3 |

**Note:** R= Retrospective, P= Prospective, CT= Clinical trial, N= number of patients, FU= follow-up, mo= months, S.I.= small intestine, L.I.= large intestine, IM = Imatinib, Preoperative IM duration per study presented as medians, mg/d = milligrams per day.

**Supplementary Table 4.** Characteristics of the included studies, their populations and interventions
